# Enhanced tomato detection in greenhouse environments: a lightweight model based on S-YOLO with high accuracy

**DOI:** 10.3389/fpls.2024.1451018

**Published:** 2024-08-22

**Authors:** Xiangyang Sun

**Affiliations:** College of Information Science and Engineering, Shandong Agricultural University, Tai’an, China

**Keywords:** greenhouse tomatoes, YOLOv8, object detection, deep learning, high accuracy, fast detection, lightweight, computer vision

## Abstract

**Introduction:**

Efficiently and precisely identifying tomatoes amidst intricate surroundings is essential for advancing the automation of tomato harvesting. Current object detection algorithms are slow and have low recognition accuracy for occluded and small tomatoes.

**Methods:**

To enhance the detection of tomatoes in complex environments, a lightweight greenhouse tomato object detection model named S-YOLO is proposed, based on YOLOv8s with several key improvements: (1) A lightweight GSConv_SlimNeck structure tailored for YOLOv8s was innovatively constructed, significantly reducing model parameters to optimize the model neck for lightweight model acquisition. (2) An improved version of the α-SimSPPF structure was designed, effectively enhancing the detection accuracy of tomatoes. (3) An enhanced version of the β-SIoU algorithm was proposed to optimize the training process and improve the accuracy of overlapping tomato recognition. (4) The SE attention module is integrated to enable the model to capture more representative greenhouse tomato features, thereby enhancing detection accuracy.

**Results:**

Experimental results demonstrate that the enhanced S-YOLO model significantly improves detection accuracy, achieves lightweight model design, and exhibits fast detection speeds. Experimental results demonstrate that the S-YOLO model significantly enhances detection accuracy, achieving 96.60% accuracy, 92.46% average precision (mAP), and a detection speed of 74.05 FPS, which are improvements of 5.25%, 2.1%, and 3.49 FPS respectively over the original model. With model parameters at only 9.11M, the S-YOLO outperforms models such as CenterNet, YOLOv3, YOLOv4, YOLOv5m, YOLOv7, and YOLOv8s, effectively addressing the low recognition accuracy of occluded and small tomatoes.

**Discussion:**

The lightweight characteristics of the S-YOLO model make it suitable for the visual system of tomato-picking robots, providing technical support for robot target recognition and harvesting operations in facility environments based on mobile edge computing.

## 1 Introduction

Tomatoes are one of the most extensively cultivated vegetables in Chinese agriculture. China not only leads globally in tomato production but also serves as a major exporter (Huo, 2016). Manual tomato harvesting requires a significant amount of labor and time. Mechanized harvesting not only cuts down on labor expenses but also boosts efficiency in the harvesting process (Li et al., 2021). Harvesting robots initially utilize computer vision systems for fruit detection, followed by guiding mechanical arms based on the detection results for harvesting operations. Therefore, fruit detection stands as a pivotal aspect throughout the entire harvesting process, with its accuracy and speed directly influencing the efficiency of harvesting robots. However, tomato fruits exhibit diverse growth postures, overlap with each other, and are heavily obscured by leaves, branches, and stems, presenting certain challenges for robot recognition. Rapid and precise identification of tomato fruits in complex greenhouse environments is a pressing issue in the development of tomato harvesting robots (Liu, 2017). Moreover, deploying models with excessively high complexity proves challenging in practical scenarios. Thus, enhancing fruit detection accuracy, speed, and lightweight improvements are crucial for bolstering the performance of harvesting robots.

Traditional methods for tomato fruit recognition in greenhouse environments rely on extracting and analyzing information based on color and shape features. Feng et al. (2015) extracted the color features of red ripe tomato fruits using the 2R-G-B color difference model and identified red ripe tomato fruits using dynamic threshold segmentation. However, this method is time-consuming and does not consider factors such as leaf occlusion in complex environments during tomato fruit recognition. Ma et al. (2016)) introduced a technique for recognizing objects by combining saliency detection with the circular random Hough transform, achieving a correct recognition rate of 77.6% for immature tomato fruits. Despite the achievements in feature design in the above studies, they suffer from slow recognition speed, low detection accuracy, and poor robustness of traditional machine vision algorithms in complex scenes, making them difficult to meet practical requirements. Although these studies have achieved certain success in feature design and tomato recognition to some extent, their slow recognition speed, low detection accuracy, and poor robustness in complex scenes cannot meet practical requirements. Additionally, they often depend on static color characteristics to recognize desired fruits. This reliance can make them less adaptable to variations in lighting and color discrepancies, resulting in reduced effectiveness when dealing with unstable color conditions. In summary, traditional methods for tomato fruit recognition fail to meet the requirements of high accuracy and real-time performance. Additionally, most of the above studies have not considered the influencing factors in complex greenhouse environments, lack robustness to diverse feature changes, and therefore, are unable to meet practical requirements.

In recent times, deep convolutional neural networks have emerged as a pivotal domain within deep learning research, attracting considerable interest. Their increasing utilization in greenhouse settings for tomato recognition has offered novel perspectives on tomato fruit identification. The detection methods of deep convolutional neural networks can be divided into two types: single-stage and two-stage detection. Region-based methods, the first type, create a set of candidate boxes and subsequently classify the targets contained within these boxes. Representative models include RCNN (Girshick et al., 2014), Fast-RCNN (Girshick, 2015), and Faster-RCNN (Ren et al., 2016). Although these methods exhibit excellent recognition accuracy with relatively low error rates and miss rates, their complex processing leads to slow detection speeds, making it difficult to meet real-time detection requirements. The second type is regression-based methods, where targets are directly classified while being located. The YOLO series networks (Redmon et al., 2016; Redmon and Farhadi, 2018; Ge et al., 2021) are typical representatives of this category. These methods have the advantage of fast recognition speed, meeting real-time requirements, and achieving accuracy levels close to the first type of methods. Given their strong real-time performance, the second type of object detection methods is beneficial for improving the efficiency of harvesting robots and monitoring devices, suitable for real-time target detection in complex environments. (Su et al. (2022)) used a lightweight YOLOv3 model in greenhouse environments, combined with lightweight networks, successfully applied it to classify tomato ripeness, achieving a 97.5% mAP. However, the model still had a large volume, making deployment challenging. Liu et al. (2020) proposed an improved tomato detection model, YOLO-Tomato, based on YOLOv3, achieving good performance. Nevertheless, the YOLOv3 model they used was large. Appe et al. (2023)) introduced a tomato detection model based on YOLOv5, which incorporates the CBAM attention mechanism into the network architecture, effectively detecting overlapping small tomatoes with an average precision of 88.1%. However, this study also faced issues with low detection accuracy. Tian et al. (2024)) proposed the TF-YOLOv5s model for detecting tomato flowers and fruits in natural environments, replacing the complete intersection over union (CIoU) loss with the efficient intersection over union (EIoU) loss and incorporating the SE attention module. Bai et al. (2024)) improved the YOLOv7 model to accurately identify strawberry seedling flowers and fruits by addressing issues such as small size, similar colors, and overlapping occlusion. They also applied the GSConv structure to optimize the model neck, achieving a 92.1% mAP with a frame rate of 45 frames per second, meeting real-time detection requirements. Li (Li et al., 2024) et al. proposed a lightweight improved YOLOv5s model for detecting dragon fruit in illuminated environments during both day and night. Meng (Meng et al., 2023) et al. proposed a spatiotemporal convolutional neural network model that utilizes a shifted window Transformer to integrate a regional convolutional neural network model for detecting pineapple fruits. Chen (Chen et al., 2024) et al. proposed a set of visual algorithms for motion target estimation, real-time self-localization, and dynamic harvesting. They also established a reliable coordination mechanism for continuous movement and picking actions. This study, inspired by previous research, addresses issues such as large model volumes, low accuracy, and difficulty in deploying actual robot vision systems. It proposes a lightweight and accurate S-YOLO model, considering tomato recognition in complex environments. Establishing a high-performance, lightweight target detection model suitable for tomato harvesting robot vision systems remains a significant challenge.

In actual greenhouse environments, tomato fruits often overlap and are heavily occluded, varying in sparsity and size, posing challenges for rapid and accurate tomato fruit recognition. Therefore, this paper introduces a novel S-YOLO model to address the aforementioned issues. This model can rapidly and accurately identify greenhouse tomato fruits while maintaining lightweight characteristics, addressing some of the limitations faced by current research and providing new technical support for the visual systems of tomato harvesting robots. This study focuses on the target detection problem for automated tomato harvesting in greenhouse environments. The core of the research is to develop and optimize a lightweight tomato target detection model, S-YOLO, aimed at enhancing the accuracy of tomato detection in complex environments. The model features high precision, a lightweight design, and rapid detection capabilities. However, the cost-effectiveness of model deployment and its practical impact on agricultural production require further discussion and analysis in future research to provide more robust support for agricultural production. This paper makes the following key contributions:

1. Introducing a S-YOLO model suitable for complex environment tomato detection, characterized by high accuracy, lightweight design, and fast speed, suitable for the visual systems of tomato harvesting robots.
2. Constructing a lightweight GSConv_SlimNeck structure suitable for YOLOv8s to optimize the model’s neck section, thereby improving model performance.
3. Creating an enhanced version of the α-SimSPPPF structure to optimize the network architecture, effectively improving detection accuracy with better performance.
4. Proposing a new enhanced version of the β-SIoU loss function, optimizing the training process, and improving tomato recognition accuracy.
5. Integrating the SE attention module into the network structure for more effective tomato feature extraction.

The paper’s structure is as follows: Section 2 covers dataset acquisition and processing. Section 3 outlines the principles of the proposed S-YOLO network structure and details improvement methods for each module. In Section 4, experimental setups are explained, and the performance of each enhanced module is thoroughly analyzed, evaluating and comparing results with other mainstream models. Finally, Sections 5 and 6 discuss and summarize the paper’s findings.

## 2 Experimental data and processing methods

### 2.1 Datasets

**Figure 1:**
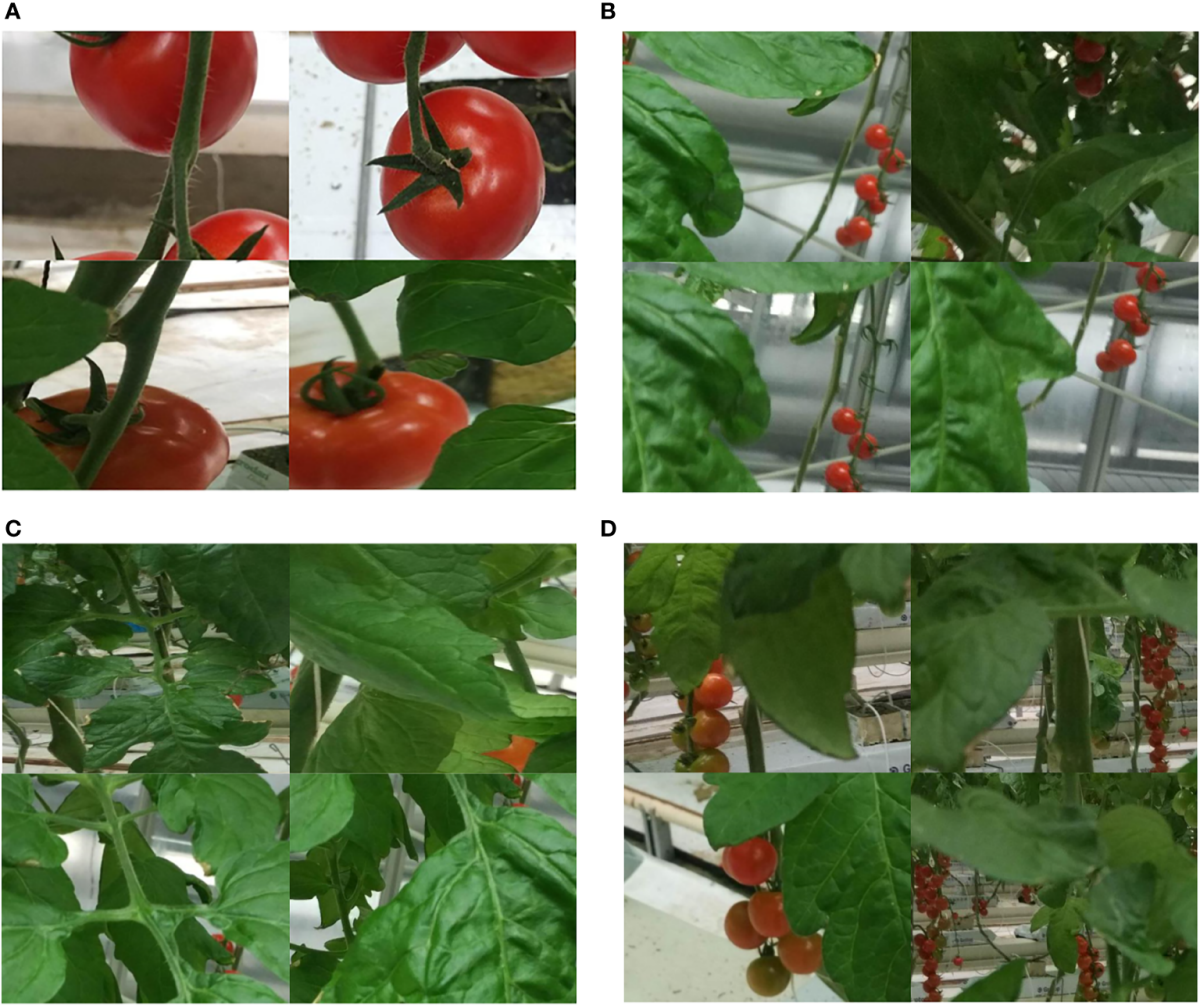
Tomato datasets. **(A)** Big tomatoes, **(B)** Small tomatoes, **(C)** Occlusion, **(D)** Clusters of tomatoes.

The dataset utilized in this research was originally obtained from the Kaggle platform, which provides resources for developers and data scientists to participate in machine learning competitions, host databases, and write and share code. The tomato dataset used in this study consists of images collected by the authors from the glass greenhouse at the National Engineering Research Center for Facility Agriculture in Chongming Base (Li et al., 2019). All images were captured in real agricultural environments, not under laboratory conditions, thus exhibiting complex backgrounds and varying brightness. The dataset comprises a total of 895 image samples. Example images from the tomato dataset in complex environments are shown in **Figure 1**, which mainly include large tomato targets, small tomato targets, occluded tomatoes, and clustered tomatoes.

### 2.2 Data preprocessing

For deep learning tasks, dataset annotation is crucial. In the case of complex greenhouse tomato images, variations in lighting conditions due to different weather and angles result in significant color differences in the collected tomato fruit images. Additionally, the diverse growth postures and severe overlapping and occlusion of greenhouse tomato fruits make it challenging to extract shape features. In this study, the LabelImg tool was used for manual annotation of tomato images, and the annotation data for each image was stored in the form of Extensible Markup Language files, following the VOC format (Everingham et al., 2010). To meet the training requirements of the detection model, the images were resized to a uniform size of 640×640 pixels and converted to RGB three-channel images. Since the YOLOv8 network incorporates online data augmentation during the training process, including techniques such as Mosaic and Mixup augmentation, and given that the dataset is not particularly small, additional offline data augmentation is generally unnecessary to save training time. Therefore, this study did not perform additional offline data augmentation.

To facilitate subsequent model training, 80% of the original 895 tomato images were allocated to the training set, 10% to the validation set, and 10% to the test set. The specific distribution is shown in **Table 1**. Finally, these datasets were utilized for training the network models, followed by additional Mixup and Mosaic data augmentation.

**Table 1:** Tomato images.

Dataset	Number
training	724
validation	81
test	90
total	895

## 3 Methods

### 3.1 Proposed S-YOLO object detection model

**Figure 2:**
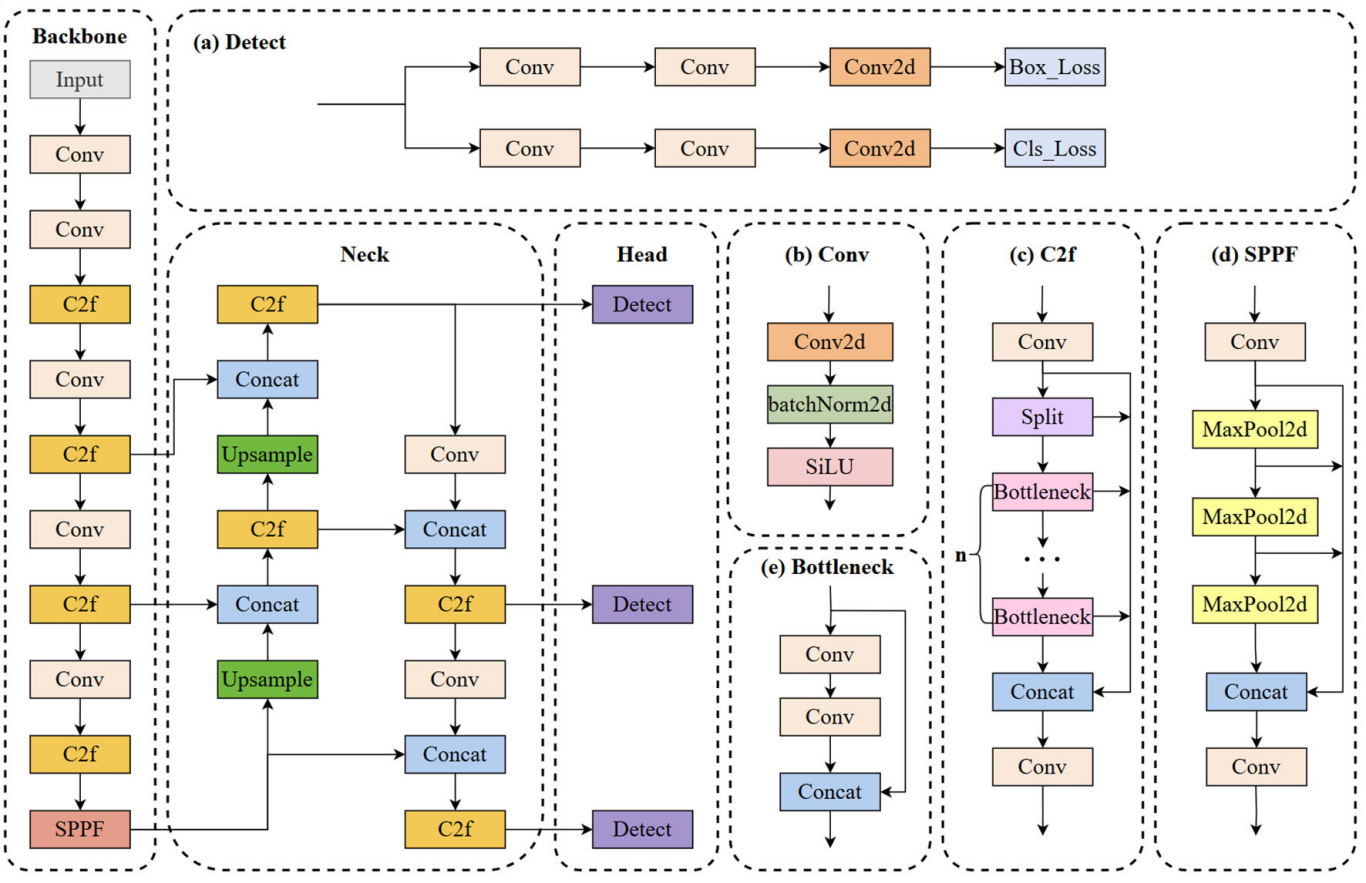
YOLOv8 algorithm model.

**Figure 2** illustrates the architecture of YOLOv8 (Reis et al., 2023). The neck and backbone parts of YOLOv8 may have drawn inspiration from the ELAN module in YOLOv7 (Wang et al., 2023). It utilizes the C2f structure to replace the C3 structure in YOLOv5 while adjusting the number of channels for various scale models. This meticulous adjustment of the model structure significantly enhances its performance. The head part adopts the current mainstream decoupled head structure, separating the classification and detection heads. It also transitions from Anchor-Based to Anchor-Free. Although the YOLOv8s model shows significant improvements, it still involves substantial computational complexity. Moreover, accurately detecting tomato fruits in complex environments remains a huge challenge.

**Figure 3:**
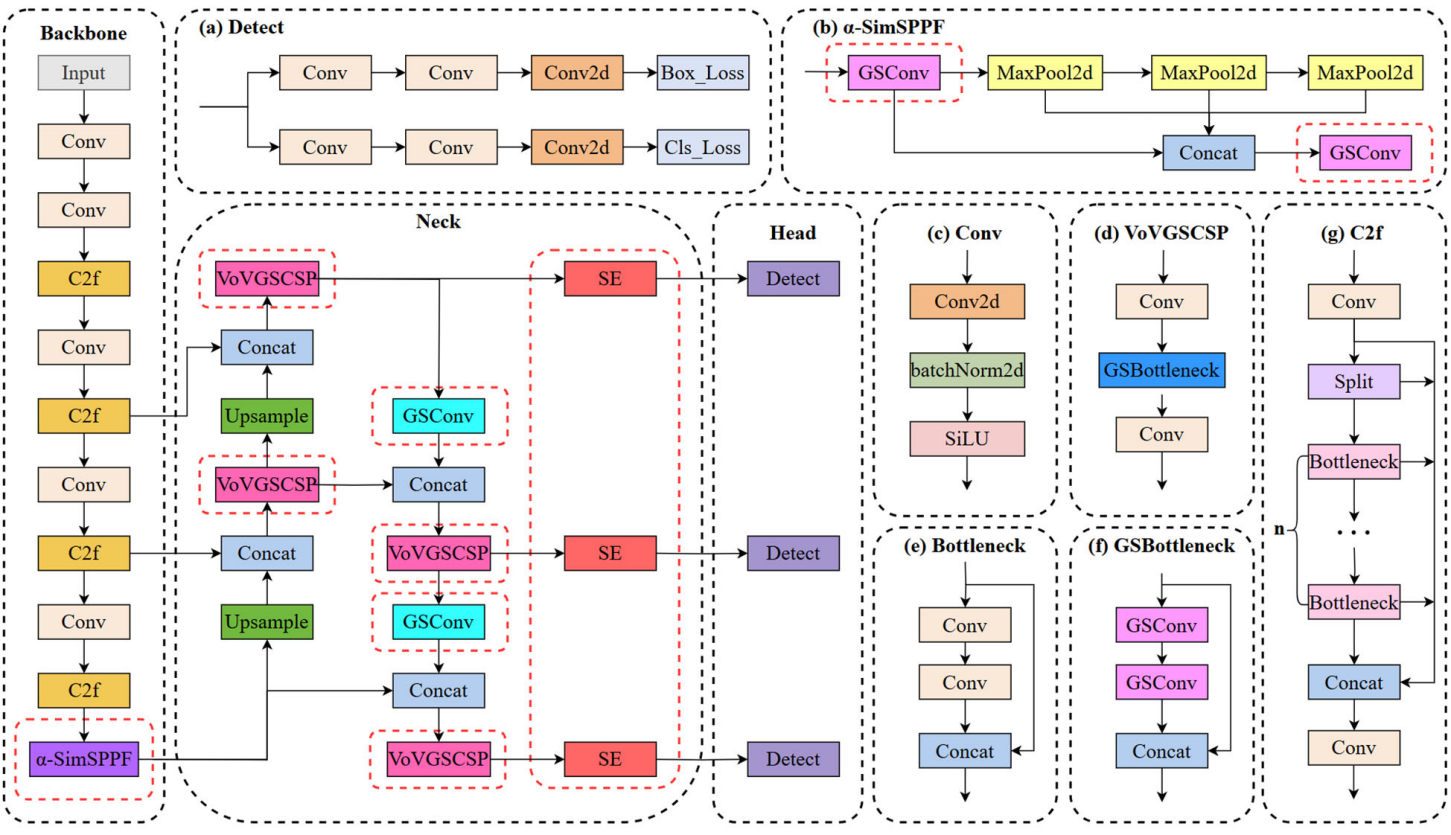
The proposed S-YOLO algorithm model. The red dashed line represents the added improvement module.

This study introduces a novel lightweight network, termed S-YOLO, which is built upon the enhancements made to the YOLOv8s architecture. This entails a meticulous optimization of the model architecture to strike a delicate balance between model complexity and performance metrics. This also involves optimizing the architecture while maximizing the model’s capability to accurately identify objects in real-time scenarios. To achieve this, four key strategies are employed. Firstly, we utilize the GSConv_SlimNeck structure to optimize the model’s neck section, effectively reducing the parameter count while ensuring performance remains intact. Secondly, we replace the original SPPF module with the newly proposed α-SimSPPF module, enhancing the model’s capabilities. Thirdly, a novel loss function, β-SIoU, is introduced to refine the training process and enhance overall model performance. Lastly, the integration of the SE attention module into the YOLOv8s’ neck network facilitates better focus on crucial features, thereby further improving the accuracy of tomato fruit target identification. **Figure 3** illustrates the architecture of the S-YOLO model proposed in this study.

### 3.2 The GSConv_SlimNeck design for YOLOv8s

**Figure 4:**
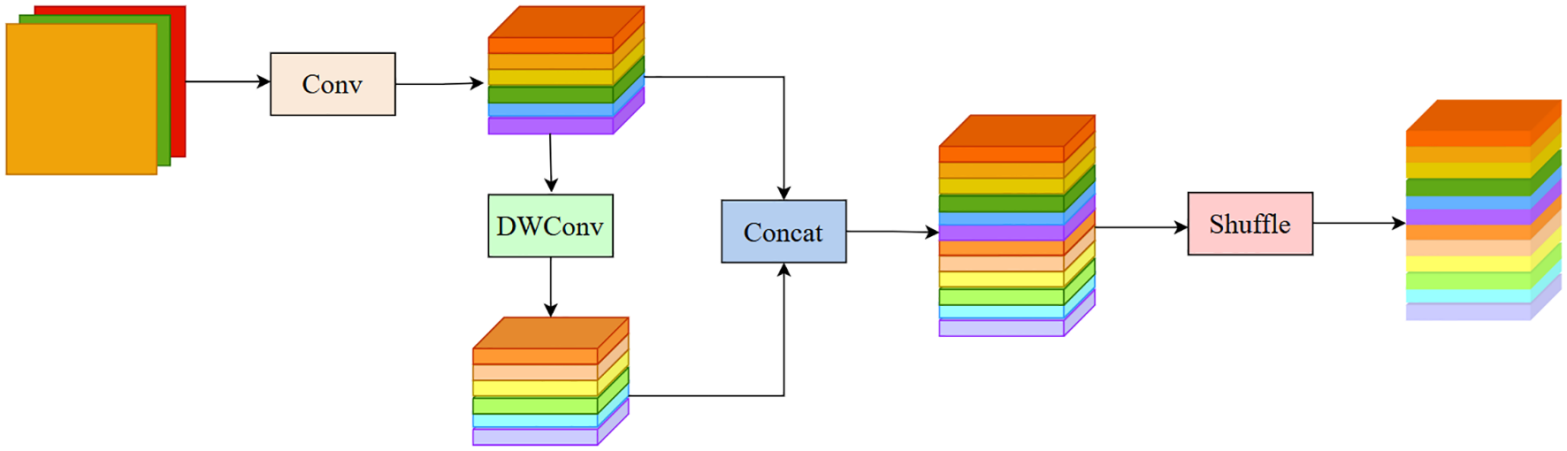
The structure of the GSConv module.

GSConv (Li et al., 2022) is a novel lightweight convolutional operation designed to reduce model complexity while maintaining accuracy. The structure of GSConv is shown in **Figure 4**. The computational cost of GSConv is approximately 60% to 70% of that of standard convolution (SC), while its contribution to model learning ability is comparable to SC. By leveraging GSConv, we can effectively utilize the advantages of Depthwise Separable Convolution (DSC) while mitigating its drawbacks on the model. SlimNeck is a design paradigm aimed at achieving higher cost-effectiveness for detectors. The core idea of SlimNeck is to use GSConv in the Neck part of the detector while maintaining a standard Backbone, which maximally reduces the impact of DSC drawbacks on the model while maintaining high accuracy. SlimNeck also introduces other modules, such as GSbottleneck and VoVGSCSP, to further improve model performance.

The original Neck structure of YOLOv8s is not sufficiently lightweight, so this paper proposes a lightweight structure, GSConv_SlimNeck, suitable for the YOLOv8s model. The construction process is as follows: Firstly, the conventional Conv structure in the Neck component is substituted with the GSConv structure. Subsequently, the terminal C2f structure within the Neck is substituted with the VoVGSCSP structure. With these two improvements, we successfully construct a lightweight GSConv_SlimNeck structure suitable for YOLOv8s, making the model more lightweight while maintaining higher detection performance.

### 3.3 The improved α-SimSPPF structure

SimSPPF is an improved spatial pyramid pooling method proposed in YOLOv6 (Li et al., 2022), which is an upgraded version of SPPF. SPPF (Spatial Pyramid Pooling Function) is a technique used for feature map pooling, commonly employed in Convolutional Neural Networks (CNNs), to pool features at different scales, thereby better capturing spatial information in images. It solves the multi-scale problem by extracting features using pooling kernels of different sizes at different scales. The fundamental concept behind SPPF involves parallel processing of the input through multiple MaxPool layers of varying sizes, followed by fusion to enhance the detector’s performance. In YOLOv5, SPPF is used to achieve feature-level fusion of local and global features. SimSPPF is an improved version of SPPF. Compared to SPPF, SimSPPF can improve the performance of the detector without increasing computational cost. SimSPPF uses ReLU activation function, while SPPF uses SiLU activation function. Structurally, SimSPPF maintains the original parallel structure of SPPF but with higher computational efficiency.

The SimSPPF structure was enhanced in this study by substituting the Conv structure with the more lightweight GSConv structure, resulting in an improved version termed α-SimSPPF. Compared to both the SPPF structure and SimSPPF, α-SimSPPF boasts higher detection accuracy with fewer parameters.

### 3.4 The enhanced β-SIoU algorithm

YOLOv8 by default utilizes the CIoU (Qiu et al., 2022) loss function, which introduces additional calculations for the distance between center points and diagonal distances. Therefore, compared to traditional IoU, the computational complexity increases, potentially adding some computational cost. CIoU’s computation method is relatively complex, requiring more processing and calculation of bounding box coordinates. Traditional methods like CIoU, DIoU (Zheng et al., 2020), etc., match IoU, center point distance, aspect ratio, etc., between real and predicted boxes but do not consider the mismatched orientation between them. This inadequacy results in slow convergence and lower efficiency, ultimately leading to poorer models.

**Figure 5:**
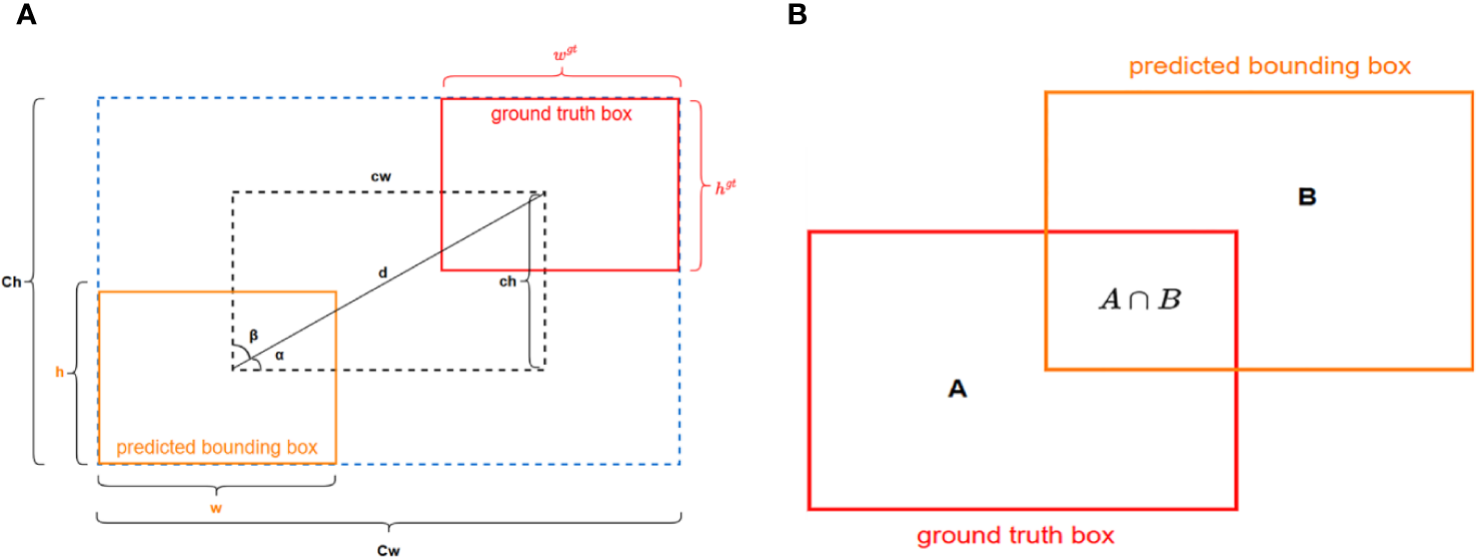
The SIoU loss function **(A)** and the IoU loss function **(B)**.

Gevorgyan (2022) proposed the SIoU loss function, which incorporates angle considerations and scale sensitivity, introducing a more complex bounding box regression method to address the limitations of previous loss functions. By integrating these aspects, better training speed and prediction accuracy can be achieved. The aim of the SIoU is to reduce the gap between predicted and actual bounding boxes, accounting for variations in shape and angle. The SIoU schematic is shown in **Figure 5**.

The process of angle loss calculation is as follows:


(1) $$Angle_{Loss}=1-2*{\text{sin}}^{2}\left(\text{arcsin}\;\left(\frac{ch}{d}\right)-\frac{\pi }{4}\right)$$

(2) $$Distance_{Loss}=2-e^{-\gamma p_{x}}-e^{-\gamma p_{y}}$$

(3) $$p_{x}={\left(\frac{cw}{Cw}\right)}^{2}$$

(4) $$p_{y}={\left(\frac{ch}{Ch}\right)}^{2}$$

(5) $$\gamma =2-Angle_{Loss}$$

In this equation, “cw” represents the disparity in width between the centers of the two bounding boxes, and “Ch” represents the height of the minimum bounding rectangle of the ground truth bounding box, while “Cw” represents the width of the minimum bounding rectangle of the predicted bounding box. The calculation process for shape loss is as follows:


(6) $$Shape_{Loss}={\left(1-e^{-W_{w}}\right)}^{\theta }+{\left(1-e^{-W_{h}}\right)}^{\theta }$$

(7) $$W_{w}=\frac{\left|w-w^{gt}\right|}{max\left(w,w^{gt}\right)}$$

(8) $$W_{h}=\frac{\left|h-h^{gt}\right|}{max\;\left(h,h^{gt}\right)}$$

In this equation, “w”, “h”, “w^gt^”, and “h^gt^” respectively represent the width and height of the predicted bounding box and the true bounding box. θ controls the emphasis on shape loss. To avoid overly focusing on shape loss and thus reducing the movement of the predicted bounding box, the authors used a genetic algorithm to compute a value close to 4. The calculation process for IoU loss is as follows:


(9) $$IoU=\frac{A\cap B}{A\cup B}$$

Where A∩B represents the intersection of the predicted bounding box and the ground truth bounding box, and A∪B represents the union of the predicted bounding box and the ground truth bounding box. The SIoU can be expressed using the following formula:


(10) $$SIoU_{Loss}=1-IoU+\frac{DistanceLoss+ShapeLoss}{2}$$

He et al. (2022) proposed the α-IoU method, which enhances bounding box regression by incorporating a power transformation into the conventional IoU loss function. Inspired by this, to bolster the robustness of SIoU towards bounding boxes and attain higher accuracy in the regression of overlapping bounding boxes, this study enhances SIoU by introducing a power of 1.5 to each of its terms. We refer to this enhanced version as β-SIoU, and its effectiveness will be demonstrated through experiments in Section 4.3.5. The computation formula is shown as follows:


(11) $$\beta -SIoU_{Loss}=1-IoU^{1.5}+{\left(\frac{DistanceLoss+ShapeLoss}{2}\right)}^{1.5}$$

### 3.5 SE attention module

Attention mechanisms facilitate models in comprehensively grasping the structure and attributes of input data, thus advancing the precision and efficiency of object detection. Attention mechanisms empower the model to discern the significance of diverse local details in the image, allowing it to concentrate more effectively on crucial features and thereby enhance the accuracy of tomato fruit detection.

The SE (Squeeze-and-Excitation) attention mechanism (Hu et al., 2018) enhances model performance by modeling the correlation between different channels. Channel-wise attention assigns different weights to different channels, focusing on channels that are crucial for recognizing specific objects. The SE module captures channel relationships through Squeeze and Excitation operations. In the Squeeze phase, it condenses the output feature map from the convolutional layer into a feature vector via global average pooling. This vector captures comprehensive statistical data from the entire feature map. During the Excitation phase, the SE module employs a fully connected layer and a nonlinear activation function to determine the significance of each channel by learning their respective weights. By incorporating Squeeze and Excitation operations, the model autonomously learns the weight and significance of individual channels, enhancing the network’s expressive power and performance. By automatically learning the weight and significance of individual channels, the network can prioritize crucial feature channels, enhancing overall model performance.

**Figure 6:**
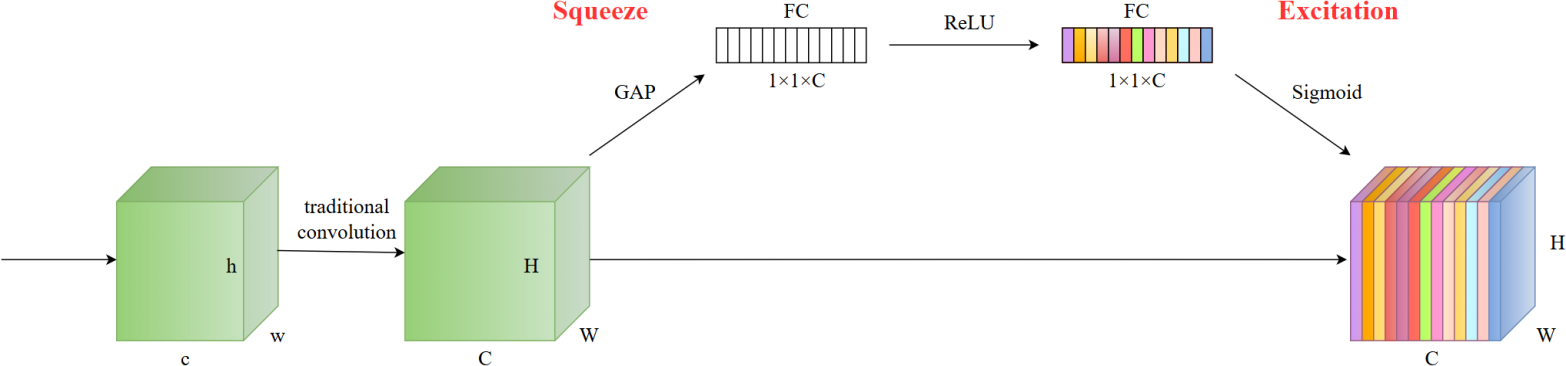
The structure of the SE attention.

After comparing different attention mechanisms, this study selected the SE attention module with the highest accuracy and incorporated it into the model’s neck. The SE attention structure is shown in **Figure 6**.

## 4 Experimental design and results analysis

### 4.1 Experimental environment and parameter setting

The experiments were conducted using PyTorch as the deep learning framework. **Table 2** provides a detailed description of the experimental setup. To optimize model training, cosine annealing was employed to update the learning rate and network weight parameters. The entire process comprised 300 iterations. The momentum factor was set at 0.937 to effectively smooth gradient updates, facilitating faster convergence and stabilizing the training process. The weight decay was set at 0.0005 to help limit the model’s complexity, prevent overfitting on the training data, and enhance the model’s generalization ability to new data. The initial learning rate was set at 0.01 to quickly reduce the loss function during the initial training phase while avoiding excessively large steps that could lead to an unstable training process. The SGD optimizer was employed, which is suitable for large deep learning models. Using the SGD optimizer simplifies the computation process, and combined with the momentum factor, effectively speeds up convergence. During the first 50 iterations, the training of the backbone network was frozen, with a batch size of 8. Freezing the backbone network’s training leverages the general features extracted by the pretrained model. This approach helps to quickly train the model with fewer computational resources and prevents disruption of the existing feature extraction capabilities. Setting the batch size to 8 improves training parallelism and efficiency within the limits of GPU memory. In the subsequent 250 iterations, the backbone network was unfrozen for training, and the batch size was adjusted to 4. Unfreezing the backbone network in the later training stage allows fine-tuning of the entire model to better adapt to the specific task’s data distribution. Adjusting the batch size to 4 helps maintain training stability and efficiency as the model complexity increases. Freezing the training is also a concept in transfer learning, as the features extracted by the neural network backbone are general. Freezing the backbone during training can accelerate the training process and prevent the weights from being disrupted.

**Table 2:** Hardware and software environment.

Configuration Item	Value
CPU	Intel i9-12900H
GPU	NVIDIA GeForce RTX 3060
CUDA	12.0
Memory	32GB
Operating system	Windows11×64
Deep learning frame	PyTorch

### 4.2 Evaluation indicators

This research selected mean Average Precision (mAP), Average Precision (AP), precision, recall, F1 score, GFLOPs, model parameters, and frames per second (FPS) as performance metrics for evaluating the deep-learning model. The evaluation metrics were calculated using the formulas below.


(12) $$Precision=\frac{TP}{TP+FN}\times 100\%$$

(13) $$Recall=\frac{TP}{TP+FP}\times 100\%$$

(14) $$F1=\frac{2\times Precision\times Recall}{Precision+Recall}\times 100\%$$

(15) $$\text{AP}=\int_{0}^{1}\text{P}\left(R\right)\text{dR}$$

(16) $$\text{mAP}=\frac{\sum_{i=1}^{n}AP_{i}}{n}$$

(17) FPS=1/T

Where TP represents the number of images where tomato fruit targets were correctly detected by the model, FP represents the number of images where non-tomato fruit targets were incorrectly detected by the model, and FN represents the number of images where tomato fruit targets were missed by the model. Precision indicates the precision rate, while Recall represents the recall rate. F1-score serves as a means to strike a balance between precision and recall. Precision and recall values are utilized to construct the precision-recall curve (PR curve), with the area under this curve denoted as AP (Average Precision). The mAP refers to the average AP. T denotes the detection time for a single image. FPS represents the number of images detected per second. The model parameters were calculated considering the input and output channel counts along with the convolutional kernel sizes, aiding in estimating the model’s size. GFLOPs are used to measure model complexity.

### 4.3 Results and analysis

#### 4.3.1 Training and validation of the S-YOLO algorithm

**Figure 7:**
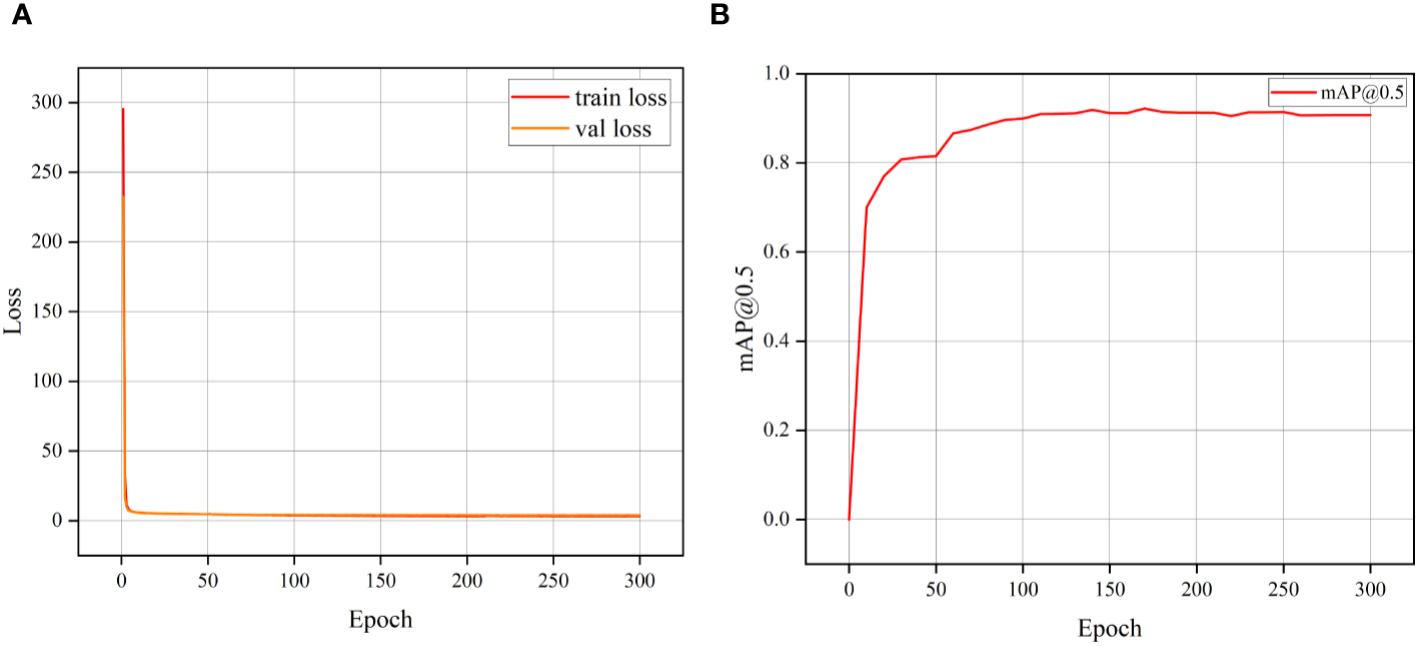
The training loss curve variation **(A)** and mAP training variation **(B)**.

**Figure 7A** displays the training loss progression of the S-YOLO algorithm. During the initial training phase, the model exhibits relatively high learning efficiency, as indicated by the rapid decline in the training loss curve, suggesting that the model is quickly learning new features. As training progresses, the rate of decrease in the loss curve gradually slows down, implying that the model is gradually stabilizing and approaching convergence. Throughout this process, both the training and validation set losses fluctuate but eventually stabilize, indicating that the model has reached the expected stable state.

In **Figure 7B**, the fluctuation of the mean Average Precision (mAP) throughout each training epoch is depicted. It can be observed that mAP rapidly increases at the beginning of training, corresponding to the rapid decline in the training loss curve. As training continues, the change in mAP stabilizes, indicating a continuous improvement in the model’s accuracy. At the 150th training epoch, mAP reaches its peak, indicating that the model is very close to its optimal performance at this point. These two figures together depict the training process of the model, from rapid learning to eventual convergence, demonstrating the effectiveness and stability of the S-YOLO model.

#### 4.3.2 Ablation experiments

We conducted ablation experiments on the tomato dataset to evaluate the performance of GSConv_SlimNeck, α-SimSPPF, β-SIoU, and SE components integrated into the model. Based on YOLOv8s, the subsequent models progressively integrated the improved modules. Model1 optimized the model’s neck structure using the GSConv_SlimNeck architecture. Model2 replaced the original SPPF structure with the enhanced version of α-SimSPPF based on Model1. Model3 introduced the proposed β-SIoU loss function on top of Model2. Ultimately, the SE attention module was embedded within the network’s neck in Model3, leading to the formulation of the S-YOLO model.

**Figure 8:**
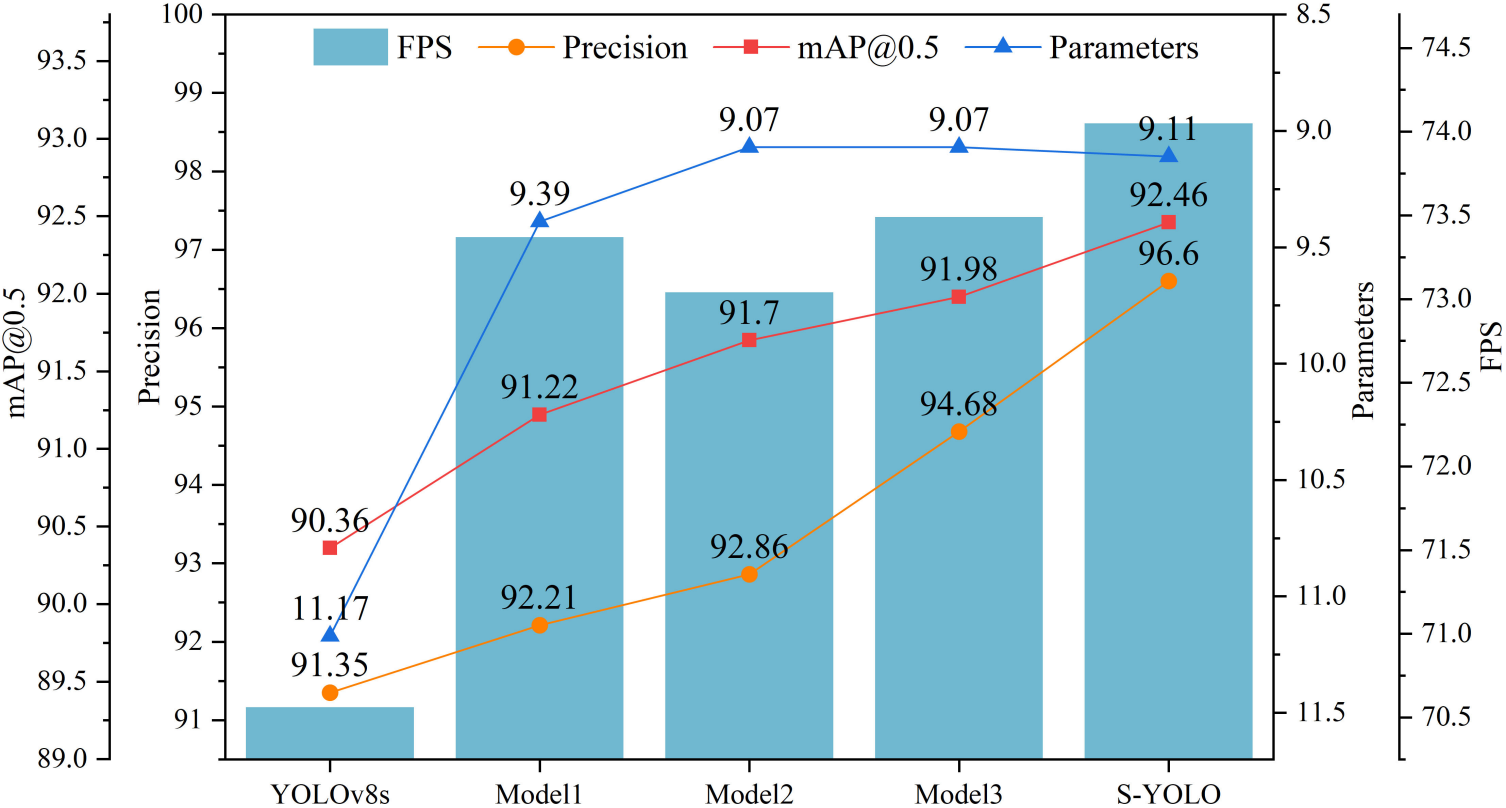
Experimental curves for different models.

As shown in **Table 3**, based on YOLOv8s, Model1 achieved improvements in several metrics by introducing the GSConv_SlimNeck structure. Precision, mAP@0.5, and FPS increased by 0.86%, 0.86%, and 2.81FPS, respectively, while model complexity and parameters decreased by 3.35G and 1.78M. The addition of the α-SimSPPF module further improved model accuracy and mAP@0.5, while reducing computational overhead. However, this improvement also slightly decreased detection speed by 0.33FPS. After adding β-SIoU to Model2, the detection rate increased by 0.45FPS compared to Model2 and exceeded YOLOv8s and Model1, compensating for the shortcomings of α-SimSPPF. This indicates a noticeable improvement in model performance with the enhanced β-SIoU loss function. The introduction of the SE attention module further improved precision, mAP@0.5, and FPS by 1.92%, 0.48%, and 0.56FPS, respectively, compared to Model3, despite a slight increase of 0.04M in model parameters. This suggests the effectiveness of the attention mechanism in extracting features relevant to tomato detection. **Figure 8** shows the experimental curves and bar charts for different models.

**Table 3:** Ablation experiments on the proposed S-YOLO algorithm.

Model	GSConv_ SlimNeck	α-SimSPPF	β-SIoU	SE	Precision	mAP@0.5	GFLOPs (G)	Parameters (M)	FPS
YOLOv8s Model1	√				91.35 92.21	90.36 91.22	28.82 25.47	11.17 9.39	70.56 73.37
Model2	√	√			92.86	91.70	25.22	9.07	73.04
Model3	√	√	√		94.68	91.98	25.22	9.07	73.49
**S-YOLO**	√	√	√	√	**96.60**	**92.46**	**25.22**	9.11	**74.05**

Bold values represent the best experimental results compared to other models.

In summary, the lightweight S-YOLO model surpasses the original YOLOv8s model significantly. Not only does it achieve model lightweighting, but it also maximizes the enhancement in detection accuracy. The model exhibits improvements across various metrics: precision, mAP@0.5, and FPS see increases of 5.25%, 2.1%, and 3.49FPS, respectively. Furthermore, the model complexity (measured in GFLOPs) and parameters are reduced by 3.6G and 2.06M, respectively, showcasing its efficiency and effectiveness in practical applications.

#### 4.3.3 Comparison of different lightweight modules

In constructing the lightweight structure GSConv_SlimNeck, both GSConv and GhostConv (Han et al., 2020) modules were compared and analyzed to validate their effectiveness. The experimental results in **Table 4** show that both GSConv and GhostConv modules contribute equally to model lightweighting, resulting in a reduction of model complexity and parameters by 3.35G and 1.78M, respectively.

**Table 4:** Experimental results for the lightweight modules.

Model	Precision	Recall	F1-Score	mAP@0.5	GFLOPs (G)	Parameters (M)	FPS
YOLOv8s	91.35	81.72	0.86	90.36	28.82	11.17	70.56
YOLOv8s + GhostConv_SlimNeck YOLOv8s + GSConv_SlimNeck	91.37 **92.21**	81.94 81.51	0.86 **0.87**	91.10 **91.22**	25.47 **25.47**	9.39 **9.39**	72.86 **73.37**

Bold values represent the best experimental results compared to other models.

However, utilizing the GSConv module to build the GSConv_SlimNeck structure exhibits superior model performance compared to using the GhostConv module. Although there is a slight decrease in recall, precision, and F1 score experience significant improvements. Specifically, compared to using GhostConv, using GSConv increases precision by 0.84%, mAP by 0.12%. Overall, the GSConv_SlimNeck structure built using GSConv demonstrates superior performance.

#### 4.3.4 Comparison of SPPF, SimSPPF, and α-SimSPPF

To verify the efficacy of the proposed α-SimSPPF structure, this study conducted a comparative analysis involving SPPF, SimSPPF, and α-SimSPPF. These three modules were placed at the same position in the model and trained accordingly. **Table 5** presents the experimental results. From various metrics, it is evident that the performance of SimSPPF is significantly lower than that of the SPPF module. However, following the enhancement from SimSPPF to α-SimSPPF, the model’s performance saw significant improvement. In comparison to the SPPF module, precision increased by 0.65% and mAP@0.5 increased by 0.48%. Additionally, the model complexity and parameters were reduced by 0.25G and 0.32M, respectively.

**Table 5:** Experimental results of SPPF, SimSPPF, and α-SimSPPF.

Model	Precision	Recall	F1-Score	mAP@0.5	GFLOPs (G)	Parameters (M)	FPS
Model1+SPPF Model1+ SimSPPF Model1+α-SimSPPF (Model2)	92.21	81.51	0.87	91.22	25.47	9.39	73.37
	91.35	81.72	0.86	91.14	25.47	9.39	72.77
	**92.86**	81.08	**0.87**	**91.70**	**25.22**	**9.07**	73.04

Bold values represent the best experimental results compared to other models.

Although using the α-SimSPPF structure resulted in a slight decrease of 0.33FPS in detection speed compared to using the SPPF structure, the accuracy and mAP@0.5 were significantly improved. Moreover, the model complexity was lower, and the model parameters were reduced, aligning with the research goal of this study. α-SimSPPF demonstrated superior performance on the dataset used in this study, with higher accuracy and lighter model, making it more suitable for tomato fruit detection and deployment in tomato harvesting robot visual systems.

#### 4.3.5 Comparison of different IoU loss functions

This study delved deeper into the influence of integrating the β-SIoU algorithm on the model’s performance, with a primary focus on comparing CIoU, DIoU, SIoU, and the β-SIoU algorithm. As shown in **Table 6**, compared to CIoU, DIoU achieved higher precision but slightly decreased mAP@0.5, while increasing the inference speed by 0.25FPS. SIoU resulted in varying degrees of decrease in precision, mAP@0.5, and FPS. However, the proposed β-SIoU algorithm demonstrated improvements across all metrics.

**Table 6:** Comparison of different loss functions.

Model	Precision	mAP@0.5	GFLOPs (G)	Parameters (M)	FPS
Model2 + CIoU	92.86	91.70	25.22	9.07	73.04
Model2 + DIoU	93.43	91.29	25.22	9.07	73.29
Model2 + SIoU	92.52	90.83	25.22	9.07	71.27
Model2 + β-SIoU(Model3)	**94.68**	**91.98**	**25.22**	**9.07**	**73.49**

Bold values represent the best experimental results compared to other models.

**Figure 9:**
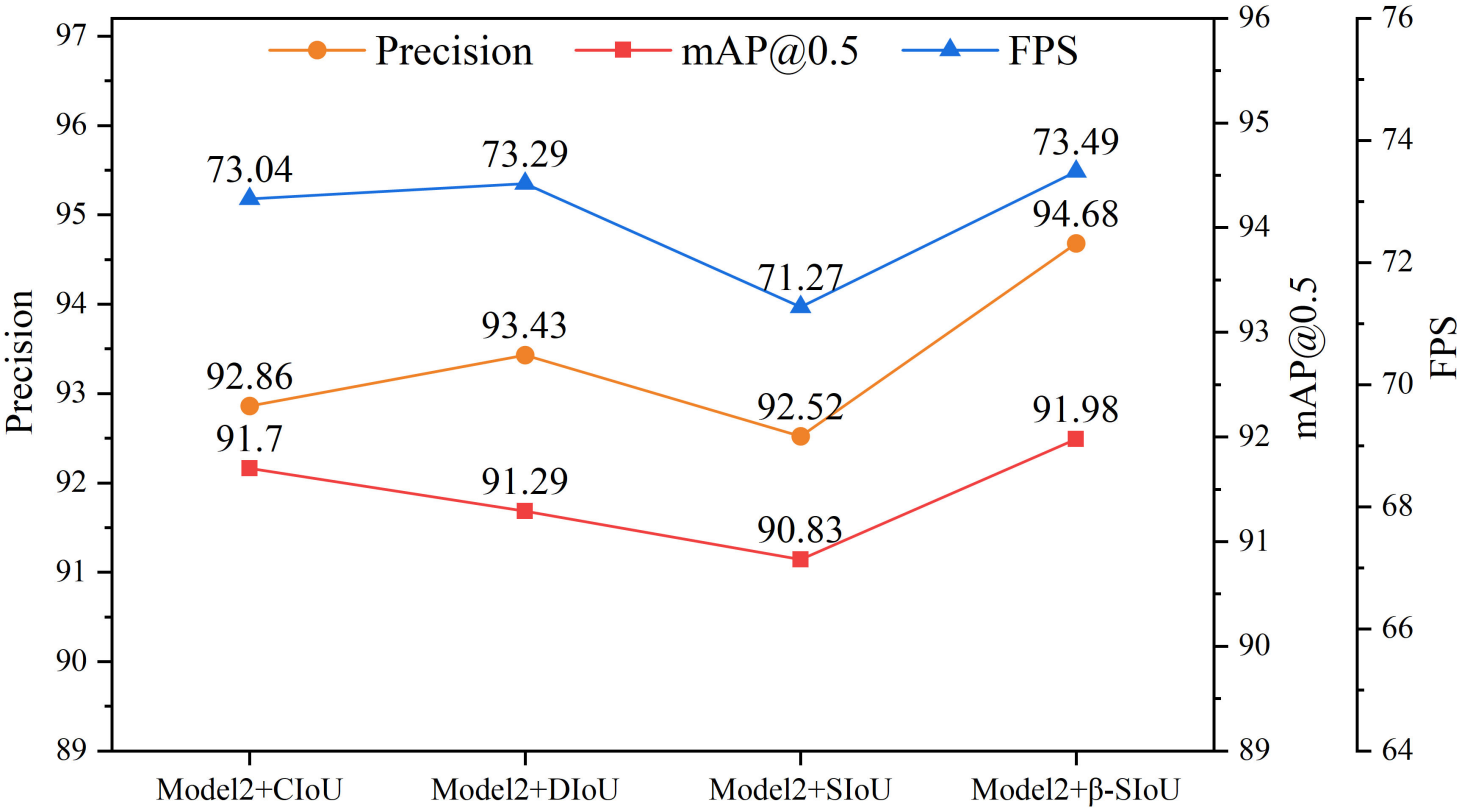
Experimental curves for different loss functions.

Among all these algorithms, Model3 stood out in multiple key metrics, particularly in precision, mAP, and processing speed. Compared to CIoU, DIoU, and SIoU, precision increased by 1.82%, 1.25%, and 2.16%, respectively, while mAP@0.5 increased by 0.28%, 0.69%, and 1.15%, respectively. Detection speed also increased by 0.45FPS, 0.2FPS, and 2.22FPS, respectively. These improvements significantly enhance model performance, making it suitable for handling overlapping and densely packed tomato objects, as well as deployment in tomato harvesting robot visual systems. **Figure 9** illustrates the experimental curves for different loss functions.

In addition, this study explored the optimal loss function for the dataset by examining different exponent values for individual terms in SIoU. As shown in **Table 7**, varying the exponent values for individual terms in SIoU had no impact on the model’s complexity. When the exponent value for individual terms in SIoU was set to 1.5, precision reached 94.68%, mAP@0.5 reached 91.98%, and the detection rate reached 73.49FPS. When each exponent in the SIoU function is set to 1.5, the model demonstrates its optimal performance.

**Table 7:** Experimental results for different exponential powers of SIoU.

Model	Exponent	mAP@0.5	GFLOPs (G)	Parameters (M)	FPS
Model2+SIoU	0.5	91.89	25.22	9.07	71.37
	1.0	90.83	25.22	9.07	71.27
	1.5	**91.98**	**25.22**	**9.07**	**73.49**
	2.0	91.23	25.22	9.07	72.03
	2.5	91.29	25.22	9.07	71.66
	3.0	90.97	25.22	9.07	72.62

Bold values represent the best experimental results compared to other models.

#### 4.3.6 Comparison of different attention modules

To delve deeper into the influence of the SE attention module and its placement within the model architecture, this study explored inserting various attention mechanisms, including ECA (Wang et al., 2020), CBAM (Woo et al., 2018), CA (Hou et al., 2021), SimAM (Yang et al., 2021), GAM (Liu et al., 2021), Shuffle (Zhang and Yang, 2021), and EMA (Ouyang et al., 2023), at the same position. Additionally, three SE attention modules were inserted into the backbone network after the third, fourth, and fifth Conv structures.

As shown in **Table 8**, adding any attention mechanism led to an improvement in accuracy. However, except for the SE attention module, which increased mAP@0.5, the other attention modules resulted in varying degrees of decrease in mAP@0.5. This suggests that the SE attention module is most suitable for incorporation into this model structure. The decrease in mAP@0.5 when adding other attention mechanisms may be due to model overfitting or neglect of certain features of tomato fruits. The SE attention module significantly improved model performance, with accuracy and mAP@0.5 increasing by 1.92% and 0.48%, respectively, compared to Model3. Moreover, the detection rate increased by 0.56FPS. Compared to Model3, adding the GAM attention module not only increased the model complexity by 15.74G and the model parameter quantity by 8.6M but also decreased mAP@0.5 and the detection rate by 1.36% and 21.51FPS, respectively, severely reducing model performance. Although the EMA attention module achieved 97.31% accuracy, both mAP@0.5 and the detection rate were significantly lower than those with the SE attention mechanism. In general, the SE attention module exhibited the most impressive performance, leading to the most substantial enhancement in the S-YOLO model’s performance.

**Table 8:** Comparison of different attention models’ performance.

Model	Precision	mAP@0.5	GFLOPs (G)	Parameters (M)	FPS
Model3	94.68	91.98	25.22	9.07	73.49
Model3 + ECA	96.10	90.94	25.22	9.07	69.30
Model3 + CBAM	95.56	91.91	25.22	9.16	69.06
Model3 + CA	95.05	90.71	25.22	9.13	73.33
Model3 + SimAM	96.01	90.83	25.22	9.07	73.62
Model3 + GAM	95.03	90.62	40.96	17.67	51.98
Model3 + Shuffle	96.67	87.17	25.22	9.07	71.47
Model3 + EMA	97.31	88.17	25.22	9.07	73.19
**Model3 + SE(S-YOLO)**	96.60	**92.46**	**25.22**	9.11	**74.05**

Bold values represent the best experimental results compared to other models.

**Figure 10:**
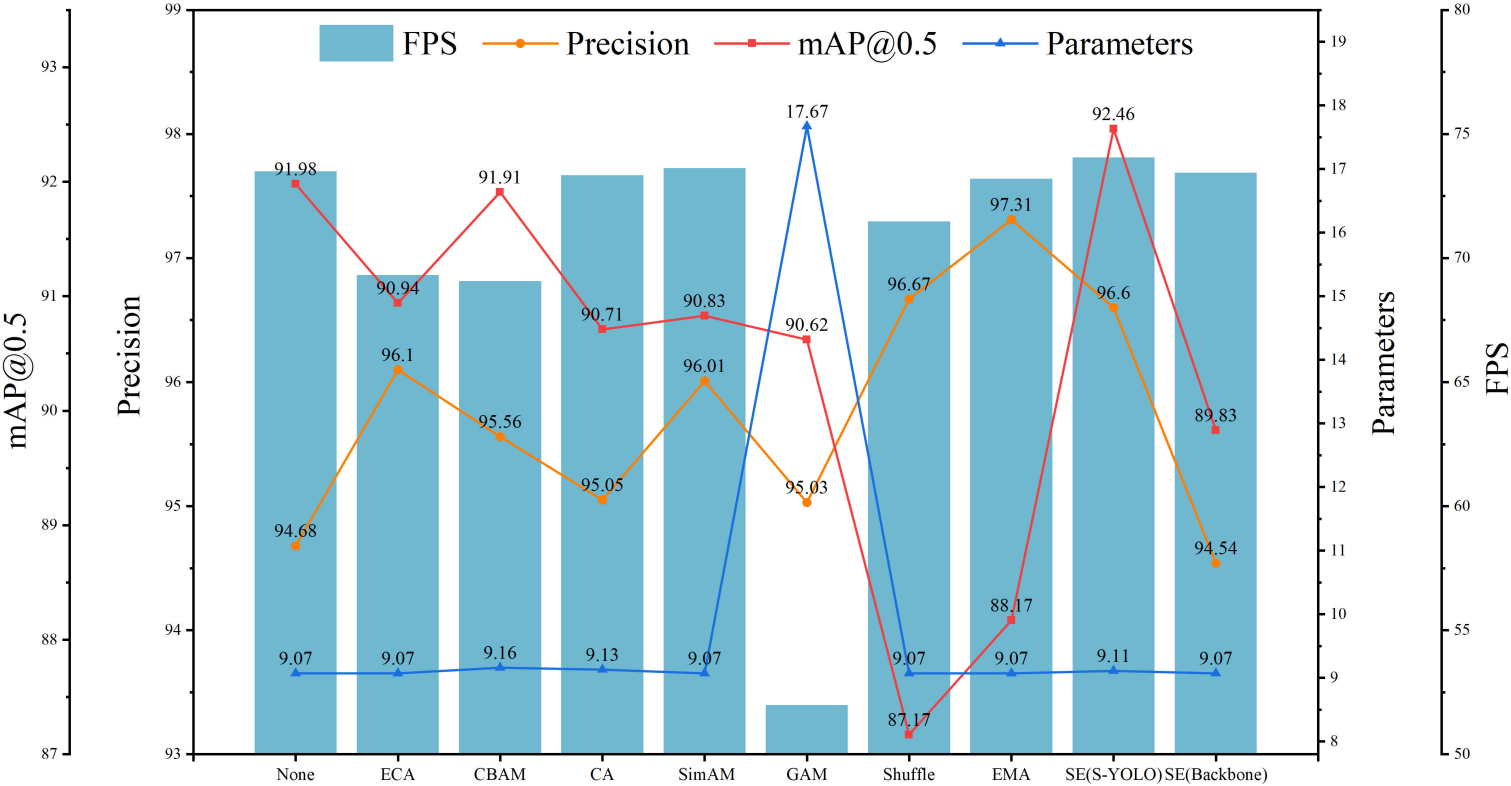
Experimental curves for different attention mechanisms.

As demonstrated in **Table 9**, incorporating the SE attention module into the backbone network resulted in a decline in model evaluation metrics. In comparison to models lacking attention mechanisms, integrating the SE attention module into the backbone network led to reductions of 0.14% and 2.15% in accuracy and mAP@0.5, respectively. However, when employing the SE attention module at the model’s neck, the accuracy and mAP@0.5 increased by 2.06% and 2.63%, respectively, compared to inserting it into the backbone network. The performance decrease resulting from inserting the module into the backbone network may be attributed to the compression of spatial and channel dimensions of the feature maps caused by introducing attention mechanisms in the backbone network. Attention mechanisms typically selectively emphasize certain features, which may lead to the neglect of other features, resulting in the loss of semantic information. This loss of information could weaken the model’s feature extraction ability. After inserting SE into the backbone network, the model’s detection speed decreased by 0.06 FPS compared to Model3, and the decrease was more significant when compared to inserting it into the neck network, reaching 0.62 FPS. **Figure 10** displays the experimental curves and bar charts for different attention modules.

**Table 9:** Experimental results on the effects of inserting attention modules at different positions.

Model	Embedding position	Precision	GFLOPs (G)	Parameters (M)	mAP@0.5	FPS
Model3	**\**	94.68	25.22	9.07	91.98	73.49
Model3 + SE	Backbone	94.54	25.22	9.07	89.83	73.43
**Model3 + SE(S-YOLO)**	Neck	**96.60**	**25.22**	**9.07**	**92.46**	**74.05**

Bold values represent the best experimental results compared to other models.

#### 4.3.7 Comparative analysis of various object-detection models’ performance

To further substantiate the model’s effectiveness, this study conducted an extensive comparison between the S-YOLO model and other prominent convolutional neural network object detection models, including the two-stage object detection model Faster RCNN, as well as the single-stage object detection algorithms CenterNet (Duan et al., 2019), YOLOv3 (Tian et al., 2019), YOLOv4 (Bochkovskiy et al., 2020), YOLOv5m (Yang et al., 2023), YOLOv7, YOLOv7x, YOLOv8m, and YOLOv8s. The experimental results are presented in **Table 10**.

**Table 10:** Comparison of different mainstream object detection models.

Model	Precision	mAP@0.5	GFLOPs (G)	Parameters (M)	FPS
Faster-RCNN CenterNet	51.42 95.88	78.42 85.14	370.21 70.22	137.10 32.67	10.57 69.77
YOLOv3	87.62	86.55	66.17	61.95	46.42
YOLOv4	66.96	72.63	60.53	64.36	36.93
YOLOv5m	88.30	86.69	51.62	21.38	44.84
YOLOv7	87.20	89.61	106.47	37.62	28.39
YOLOv7x	91.56	88.84	190.58	71.34	17.94
YOLOv8m	93.19	91.69	79.32	25.90	37.57
YOLOv8s	91.35	90.36	28.82	11.17	70.56
**S-YOLO**	**96.60**	**92.46**	**25.22**	**9.11**	**74.05**

Bold values represent the best experimental results compared to other models.

Faster RCNN is a typical two-stage object detection algorithm, but its model size is large, with model complexity and parameters much higher than other single-stage object detection algorithms. Its detection speed is only 10.57 FPS, which is only 14.27% of S-YOLO’s. The model complexity is as high as 370.21G, about 15 times that of S-YOLO, and the model parameters are as high as 137.10M, about 14 times that of S-YOLO. S-YOLO’s accuracy, mAP@0.5, and FPS are 45.18%, 14.04%, and 63.48FPS higher than Faster RCNN, respectively. Overall, the performance of the S-YOLO model far exceeds that of Faster RCNN.

**Figure 11:**
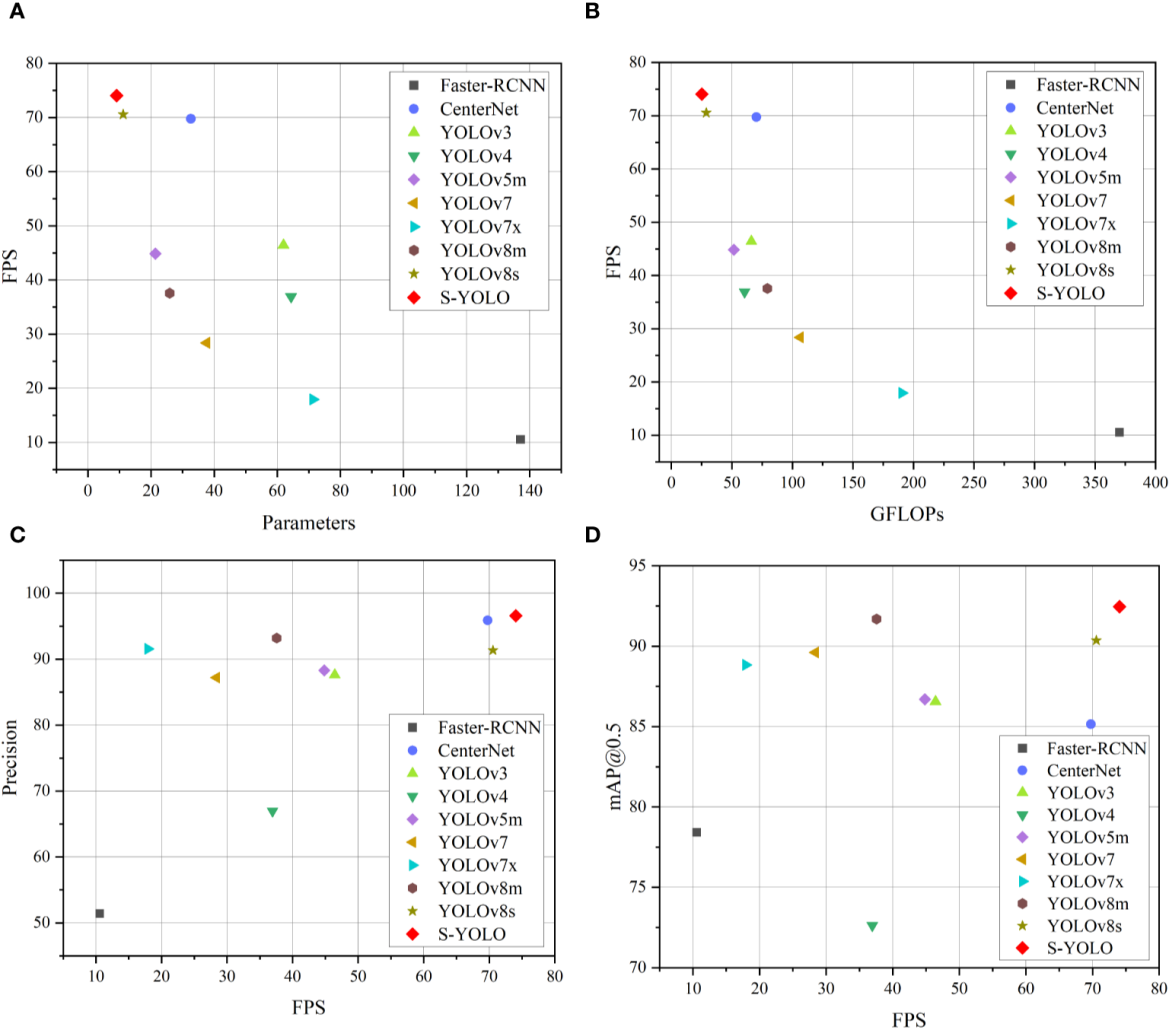
Scatter plots of the experiments for different models. **(A)** FPS-Parameters, **(B)** FPS-GFLOPs, **(C)** Precision-FPS, **(D)** mAP@0.5-FPS.

In comparison to other models, S-YOLO outperforms other models across all metrics. The model accuracy, mAP@0.5, and detection speed are 96.60%, 92.46%, and 74.05FPS, respectively, with model complexity and parameters of only 25.22G and 9.11M. Compared to CenterNet, the S-YOLO model shows advantages in mAP@0.5, model complexity, model parameters, and FPS, with mAP@0.5 7.32% higher, FPS 4.28FPS higher, and model complexity and parameters only 35.91% and 27.88% of CenterNet, respectively. YOLOv3 and YOLOv5m have similar model complexities and detection speeds, but their overall performance is much lower than S-YOLO. YOLOv4 has the lowest accuracy and mAP@0.5 among all models. Due to the higher model complexity of YOLOv7, YOLOv7x, and YOLOv8m, they also have a certain impact on detection speed, which is 45.66FPS, 56.11FPS, and 36.48FPS lower than S-YOLO, respectively, indicating that the lightweight improvements of S-YOLO have a certain effect on improving detection speed. Compared to the YOLOv8s model, the S-YOLO model has higher accuracy by 5.25%, mAP@0.5 by 2.1%, and FPS by 3.49FPS, with model complexity and parameters reduced by 3.6G and 2.06M, respectively, indicating that the improved S-YOLO model has improved in all indicators, and the model performance has been significantly improved. The following **Figure 8** provides a more intuitive illustration of the unique advantages of S-YOLO compared to other models, achieving the optimal balance between model detection speed, lightweight, and accuracy. **Figure 11** illustrates that the S-YOLO model excels over other models in various aspects.

In summary, the S-YOLO model performs significantly better than current mainstream object detection models, with high accuracy while being lightweight, providing technical references for the deployment of tomato harvesting robot vision systems.

#### 4.3.8 Model visualization results

**Figure 12:**
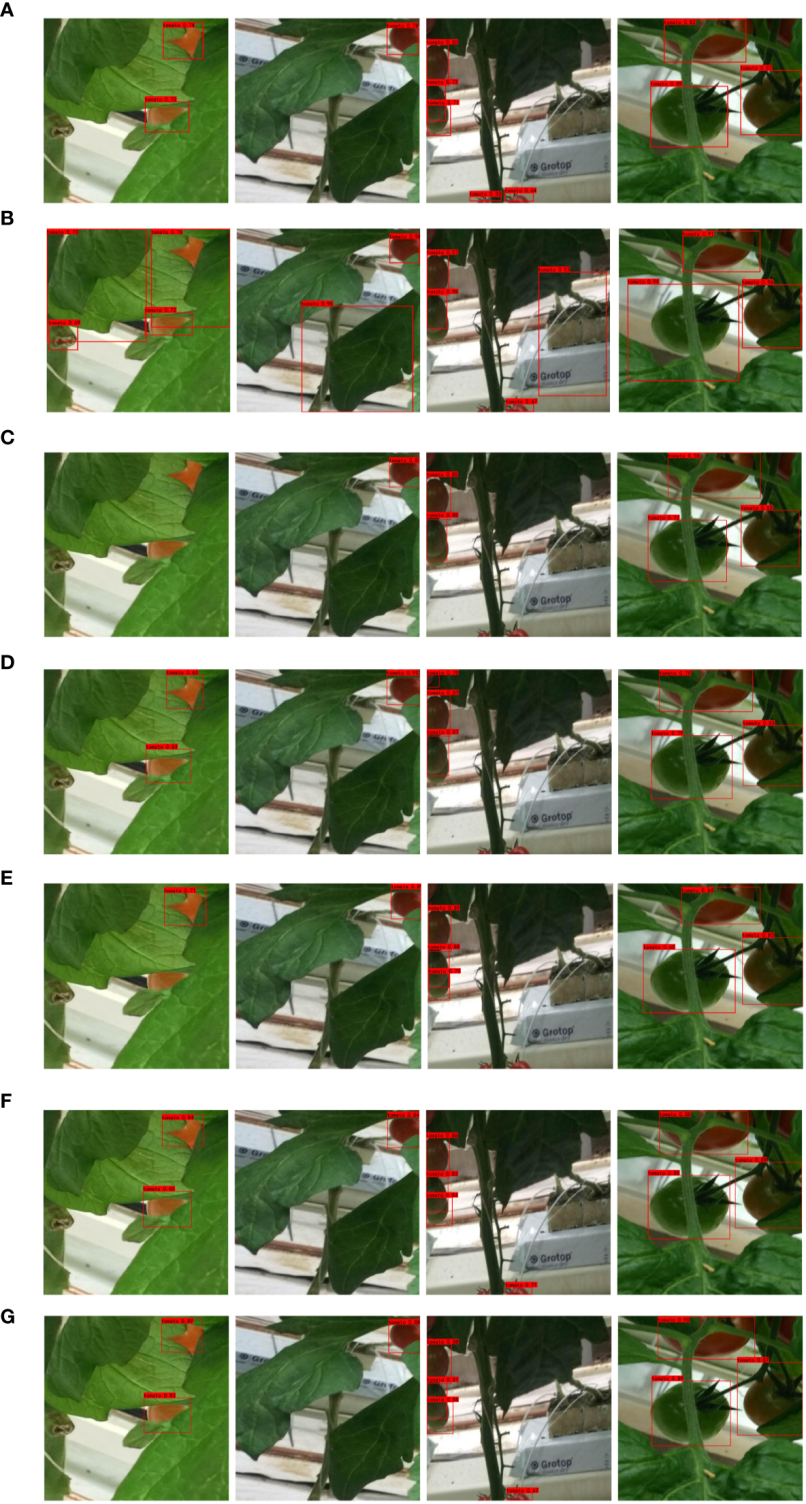
Visual detection comparison results of different models. **(A)** CenterNet, **(B)** YOLOv4, **(C)** YOLOv5m, **(D)** YOLOv7, **(E)** YOLOv7x, **(F)** YOLOv8s, **(G)** S-YOLO.

The detection performance of CenterNet, YOLOv4, YOLOv5m, YOLOv7, YOLOv7x, YOLOv8s, and S-YOLO models is illustrated in **Figure 12**. For the YOLOv4 model, there are numerous detection errors, incorrectly identifying tomato leaves and other objects as tomato fruits. The YOLOv5m model exhibits poor detection performance for occluded tomatoes, resulting in missed detections and overall poor recognition. YOLOv7x also struggles with accurately detecting occluded tomatoes. The overall detection accuracy of CenterNet, YOLOv7, and YOLOv8s is lower than that of the S-YOLO model, with S-YOLO achieving higher accuracy overall. In summary, the S-YOLO model not only achieves lightweight design but also significantly outperforms other models in tomato fruit detection.

## 5 Discussion

This study investigates an improved lightweight S-YOLO model designed for accurately detecting tomato fruits in greenhouse environments, including occluded and small target tomatoes. It provides a technical reference for the visual system of tomato harvesting robots, addressing issues such as low detection efficiency and accuracy, thus holding considerable practical value.

Previous research has shown limitations in terms of accuracy, lightweight design, or detection speed. In this work, a lightweight GSConv_SlimNeck structure is constructed to optimize the model’s neck region. To enhance detection accuracy, the α-SimSPPF structure and β-SIoU loss function are proposed. Additionally, the incorporation of the SE attention module enhances the accuracy of the model. By implementing these enhancements, the proposed S-YOLO model significantly outperforms other object detection models, achieving substantially improved accuracy in tomato detection while maintaining lightweight characteristics. Ultimately, the S-YOLO model achieves 96.60% accuracy, 92.46% mAP@0.5, with a parameter count of only 9.11M and a detection speed of 74.05FPS, demonstrating excellent detection performance.

While this study has made progress in tomato detection in greenhouse environments, there are still limitations to address. For instance, the proposed model may face significant limitations in detection speed when running on low-cost devices. Considering the cost limitations of harvesting robot hardware and the pressing need for real-time detection, future studies should prioritize further size reduction of the model to expedite its processing speed. This will ensure real-time tomato detection and enhance its suitability for integration into the visual systems of tomato harvesting robots.

## 6 Conclusions

This study introduces a novel model named S-YOLO, characterized by its lightweight design and exceptional accuracy. It effectively addresses the low accuracy in detecting occluded and small tomatoes, providing technical guidance for the visual systems of tomato harvesting robots. Through experimental research and result analysis, the main contributions can be summarized as follows:

1. Lightweight Design: A GSConv_SlimNeck structure suitable for YOLOv8s is constructed to optimize the model’s neck region, achieving model lightweightness.
2. Accuracy Improvement: The substitution of the SPPF module with the upgraded α-SimSPPF structure and the replacement of the CIoU loss function with the enhanced β-SIoU loss function contributed to the improved accuracy of the model’s detection capabilities.
3. Effective Feature Extraction: Additional SE attention module is introduced to focus on crucial information, further enhancing feature extraction for occluded and small target tomatoes.

Compared to traditional object detection algorithms, S-YOLO demonstrates robustness, lightweight design, and outstanding detection performance, providing technical support for efficiently identifying tomato fruits in tomato harvesting robots. In the future, more tomato fruit images captured in greenhouse environments will be collected, and the model will be further improved in a more lightweight manner to provide stronger technical support for the visual systems of tomato robots.

## Data Availability

The original contributions presented in the study are included in the article/supplementary material. Further inquiries can be directed to the corresponding author.
